# A high-quality genome for the slender anole (*Anolis apletophallus*): an emerging model for field studies of tropical ecology and evolution

**DOI:** 10.1093/g3journal/jkad248

**Published:** 2023-10-24

**Authors:** Renata M Pirani, Carlos F Arias, Kristin Charles, Albert K Chung, John David Curlis, Daniel J Nicholson, Marta Vargas, Christian L Cox, W Owen McMillan, Michael L Logan

**Affiliations:** Department of Biology, University of Nevada Reno, Reno, NV 89557, USA; Smithsonian Tropical Research Institute, Panama City 0843-03092, Panama; Smithsonian Tropical Research Institute, Panama City 0843-03092, Panama; Data Science Lab, Office of the Chief Information Officer, Smithsonian Institution, Washington, DC 20013, USA; Department of Biology, University of Nevada Reno, Reno, NV 89557, USA; Smithsonian Tropical Research Institute, Panama City 0843-03092, Panama; Department of Ecology and Evolutionary Biology, Princeton University, Princeton, NJ 08544-2016, USA; Smithsonian Tropical Research Institute, Panama City 0843-03092, Panama; Department of Ecology and Evolution, University of Michigan, Ann Arbor, MI 48109-1085, USA; Smithsonian Tropical Research Institute, Panama City 0843-03092, Panama; University of Texas, Arlington, TX 76019, USA; Smithsonian Tropical Research Institute, Panama City 0843-03092, Panama; Smithsonian Tropical Research Institute, Panama City 0843-03092, Panama; Department of Biological Sciences and Institute of Environment, Florida International University, Miami, FL 33199, USA; Smithsonian Tropical Research Institute, Panama City 0843-03092, Panama; Department of Biology, University of Nevada Reno, Reno, NV 89557, USA; Smithsonian Tropical Research Institute, Panama City 0843-03092, Panama

**Keywords:** adaptive radiation, *Anolis*, bioinformatics, genetics, genome, molecular evolution, Panama

## Abstract

The slender anole, *Anolis apletophallus*, is a small arboreal lizard of the rainforest understory of central and eastern Panama. This species has been the subject of numerous ecological and evolutionary studies over the past 60 years as a result of attributes that make it especially amenable to field and laboratory science. Slender anoles are highly abundant, short-lived (nearly 100% annual turnover), easy to manipulate in both the lab and field, and are ubiquitous in the forests surrounding the Smithsonian Tropical Research Institute in Panama, where researchers have access to high-quality laboratory facilities. Here, we present a high-quality genome for the slender anole, which is an important new resource for studying this model species. We assembled and annotated the slender anole genome by combining 3 technologies: Oxford Nanopore, 10× Genomics Linked-Reads, and Dovetail Omni-C. We compared this genome with the recently published brown anole (*Anolis sagrei*) and the canonical green anole (*Anolis carolinensis*) genomes. Our genome is the first assembled for an *Anolis* lizard from mainland Central or South America, the regions that host the majority of diversity in the genus. This new reference genome is one of the most complete genomes of any anole assembled to date and should facilitate deeper studies of slender anole evolution, as well as broader scale comparative genomic studies of both mainland and island species. In turn, such studies will further our understanding of the well-known adaptive radiation of *Anolis* lizards.

## Teaser

Several life history features of the Panamanian slender anole have made it the focus of ecological and evolutionary study since the 1960s; however, lack of a reference genome has limited the depth of research. Here, Pirani et al. report an annotated, high-quality, and highly contiguous genome assembled through multiple rounds of improvement. Comparison of the slender anole genome to other anole genomes uncovers an interesting aspect to its evolution.

## Introduction

Lizards of the genus *Anolis* have played a significant role in our understanding of adaptive radiation (Losos 2009). They represent a hyperdiverse vertebrate genus composed of ∼400 species that are distributed throughout southeastern North America and most of Latin America, as well as the Caribbean (Losos 2009). Famously, entire communities composed of similar sets of ecomorphs have repeatedly evolved across the Greater Antillean islands of the Caribbean, providing a classic example of convergent evolution (Losos 2009). Research on anole ecomorphs has led to important discoveries on how processes like competition and character displacement can affect community assembly and structure (Losos 1992, 1995). This combination of high diversity, rapid evolution, and replication of evolutionary outcomes has also made anoles important model organisms for the study of speciation and contemporary evolution (Losos *et al.* 2004, 2006; Roger *et al.* 2008; Logan *et al.* 2014; Lapiedra *et al.* 2018; Calsbeek *et al.* 2022). The high natural abundances of anoles and their tractability for both field observation and laboratory study have made them one of the most important vertebrate models for ecological (Schoener *et al.* 2002; Pringle *et al.* 2019), behavioral (Johnson *et al.* 2010), and ecophysiological research (Huey and Webster 1976). Finally, anoles have colorful throat fans called dewlaps which they use to communicate with con- and heterospecifics, and this group has therefore been a major focus of studies on signal evolution as well (Fitch and Hillis 1984; Losos 1985; Leal and Fleishman 2004; Nicholson *et al.* 2007; Ng *et al.* 2017).

Despite the rich history of research on anoles, high-quality genomes have only been published for a few species (Alföldi *et al.* 2011; Tollis *et al.* 2018; Geneva *et al.* 2022; Kanamori *et al.* 2022). Of these, the genomes of the green anole (*Anolis carolinensis*) and brown anole (*Anolis sagrei*) are the most complete (Alföldi *et al.* 2011; Geneva *et al.* 2022). The green anole genome was assembled and annotated more than a decade ago and has been the foundation of many investigations, including those on differences in gene expression under natural selection (Campbell-Staton *et al*. 2022) and local adaptation to extreme environments (Campbell-Staton *et al.* 2018). Unfortunately, nearly all available genomic resources come from West Indian and Caribbean species, with only a few relatively low-quality genomes published to date for Central and South American species (including a previous, incomplete genome for the slender anole; Tollis *et al.* 2018), even though species diversity is highest in this region.

**Fig. 1.**
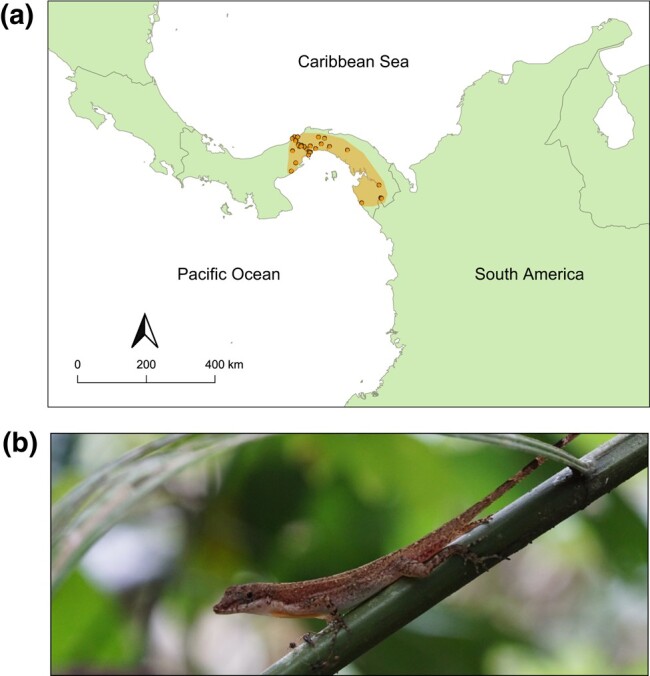
a) The distribution and georeferenced records in Panama (orange dots; data downloaded from gbif.org in 2022 according to (Köhler and Sunyer 2008) for the slender anole (*A. apletophallus*). b) A male slender anole in situ (photo: RMP).

Here, we assemble and annotate a high-quality genome for the slender anole, *Anolis apletophallus* (Köhler and Sunyer 2008), to facilitate further research on this species and anoles in general. The slender anole is part of the *Norops* species group and was previously thought to represent central and eastern Panamanian populations of *Anolis limifrons* (Nicholson *et al.* 2012), which is a common anole with a broad distribution in Central America, until the 2 species were distinguished from each other based on substantial differences in the morphology of the hemipenes (Köhler and Sunyer 2008). Like *A. limifrons*, the slender anole is an arboreal lizard of the lowland rainforest understory (see Fig. 1). This lizard is small in body size (adults are 40–45 mm in snout–vent length), lays multiple single-egg clutches primarily during the rainy season (April–November), and displays rapid growth to maturity with high adult mortality (Andrews and Nichols 1990) that results in annual population turnover (Andrews *et al.* 1982; Andrews and Rand 1983). Additionally, slender anoles are extremely abundant in the forests surrounding the research facilities of the Smithsonian Tropical Research Institute (Cox *et al.* 2020). These features of slender anole geography and biology, in combination with their tractability for laboratory research, have inspired 6 decades of work on this species. Studies on slender anoles in Panama have contributed to a range of subfields within evolutionary ecology, including population ecology (Sexton 1967; Andrews 1991; Stapley *et al.* 2015; Andrews and Rand 2022), morphological evolution (Andrews and Stamps 1994), invasion biology (Nicholson *et al.* 2022), life history evolution (Andrews *et al.* 1989), microbial ecology (Kimsey 1992; Williams *et al.* 2022), species interactions (Chalcraft and Andrews 1999), ecophysiology (Logan *et al.* 2021; Neel *et al.* 2021), and signal evolution (Stapley *et al.* 2011; Rosso *et al.* 2020). A well-annotated reference genome would greatly benefit future research on this species.

## Materials and methods

### Specimen, library construction, and sequencing of the slender anole genome

We collected an adult female slender anole from the wild (Soberanía National Park, Panama; Fig. 1a) and stored it at −80°C at the Smithsonian facility on Naos Island (collection permit: SE/A-45-2020 issued by MiAmbiente). We used a female because they are likely the homogametic sex in slender anoles, and this reduced the likelihood of assembly problems caused by unmatched sex chromosomes. We extracted high molecular weight (HMW) DNA from frozen muscle tissue using a phenol–chloroform method (Barnett and Larson 2012). We purified the extracted DNA with 3× concentration of KAPA Pure Beads (Roche Sequencing) and quantified the purity using a Qubit fluorometer (Thermo Fisher Scientific). We also checked for the integrity of the DNA with a field inversion gel (Pippin Pulse, Sage Science). On average, DNA fragments were ∼50 kb in length.

Sequencing was performed by combining 3 technologies: Oxford Nanopore (ONT, Oxford, United Kingdom), 10× Genomics Linked-Reads (10× Genomics Chromium platform), and Dovetail Omni-C (Hi-C). We prepared the ONT libraries using the ligation sequencing kit SQK-LSK109 according to the manufacturer's protocols. ONT libraries started with 1.5 μg of HMW DNA, and genomic DNA was first fragmented to ∼10 kb using Covaris g-TUBEs following the manufacturer's protocols. We end-repaired the fragmented DNA using a NEBnext Ultra II End Repair kit (New England Biolabs) and cleaned it with 1× volume of KAPA Pure Beads (Roche sequencing). Next, we performed adapter ligations for 15 min using NEB blunt/TA ligase (New England Biolabs). The libraries were cleaned using 0.4× AmPure beads (Beckmann Coulter) and eluted in 15 μL of elution buffer. Subsequently, we performed sequencing on a MinION Mk1b (Oxford Nanopore) using SpotON flow cells (FLO-MIN106; Oxford Nanopore) in 48-h sequencing runs controlled and monitored by the MinKNOW software (release 19.06.8, Oxford Nanopore). We generated and saved the FAST5 files during sequencing and performed base calling using Guppy (v.3.3.0; Oxford Nanopore). We used Porechop (v.0.2.3, https://github.com/rrwick/Porechop) to remove residual ONT adapters and NanoFilt (v.2.5; https://github.com/wdecoster/nanofilt; De Coster *et al.* 2018) to filter reads with an average quality score > Q5. For visualization, we used NanoPlot (v.1.10; De Coster *et al.* 2018) to graph ONT read qualities.

HMW DNA extracted from the same individual was used for generating the ONT libraries, and this DNA was also used for 10× Genomics Linked-Reads library preparation. A total of 1.5 μg of HMW DNA was loaded onto a Chromium Controller chip with 10× Chromium reagents and gel beads, following the manufacturer's protocols. We sequenced barcoded DNA fragments on an Illumina Hiseq X Ten platform (Illumina HiSeq X Ten, RRID:SCR_016385) to yield 2 × 150 bp paired-end sequences. Library preparation and sequencing were performed at Genome Quebec (McGill University and Génome Québec InnovationCentre). We checked quality of the raw 10× Illumina short reads with FastQC (v.0.11.8; Andrews 2010), and adapters were trimmed with the Cutadapt program (v.3.4; Martin 2011). We estimated genome size, heterozygosity, repeat, and duplicate content on clean and filtered 10× Genomics Linked-Reads using GenomeScope (https://github.com/schatzlab/genomescope; Vurture *et al.* 2017). We counted and generated a *k*-mer frequency distribution for 21-mers with Jellyfish (v.2.2.6; https://github.com/gmarcais/Jellyfish), and the resulting histogram was later processed by GenomeScope (Marcais and Kingsford 2011).

Finally, we sent HWM DNA from a different female individual (collected from the same site as the previous female) to Dovetail Genomics (Scotts Valley, CA), who performed all subsequent library construction, sequencing, assembly, and scaffolding. The Hi-C library was constructed following the methods described by Putnam *et al.* (2016). Briefly, chromatin was fixed in place with formaldehyde in the nucleus and then extracted. Fixed chromatin was digested with DNase I, and chromatin ends were repaired and ligated to a biotinylated bridge adapter followed by proximity ligation of adapter-containing ends. After proximity ligation, crosslinks were reversed, and the DNA was purified. Purified DNA was treated to remove biotin that was not internal to ligated fragments. Sequencing libraries were generated using NEBNext Ultra enzymes and Illumina-compatible adapters. Biotin-containing fragments were isolated using streptavidin beads before PCR enrichment of each library. Libraries were sequenced on an Illumina HiSeqX platform to 20× coverage.

### Hybrid de novo genome assembly of the slender anole genome

We used a hybrid genome assembly pipeline to combine Oxford Nanopore (ONT), 10× Genomics Linked-Reads technologies, and Hi-C Dovetail proximity ligation data. First, we assembled a draft genome from ONT long reads using Wtdbg2 (v2.2; Ruan and Li 2020). Wtdbg2 is a fast de novo assembler for noisy long-read sequence data produced by ONT technologies (see https://github.com/ruanjue/wtdbg2; Ruan and Li 2020). We ran Wtbg2 with the “-ont” preset option as suggested by Ruan and Li (2020). ONT reads were used to polish the contigs by mapping the reads with Minimap2 (Li 2018). This was followed by a round of polishing with the 10× Genomics Linked-Reads, which we mapped to the assembly with BWA (Walker *et al.* 2014). Similar to the polishing with ONT reads, we obtained a consensus assembly with the wtpoa-cns command from Wtdbg2 (v. 2.2; Ruan and Li 2020). Additionally, we performed a medium-range scaffolding with 10× Genomics Linked-Reads using Scaff10× software (v.4.2; https://github.com/wtsi-hpag/Scaff10X). A further round of polishing was performed with both ONT data with Racon (v.1.4.20; Vaser *et al.* 2017) and 10× reads with Pilon (v.1.23; Walker *et al.* 2014). At this stage, we checked and eliminated bacterial, viral, and plasmid contamination using Kraken2 (v.2.1.2; https://github.com/DerrickWood/kraken2). This process resulted in 15,176 sequences rooted (91.08%) and 1,487 sequences unclassified (8.92%). To filter the contigs, we made them a target in the genome. Subsequently, we created a list using the human and unclassified contigs and removed any contigs classified as bacteria, plasmids, or viruses, which resulted in the deletion of 613 contigs. Using Samtools, we applied the created list to extract only the contigs identified as human (vertebrate) or unclassified from the genome assembly.

To improve the contiguity of the slender anole genome, we used HiRise Pipeline, a software platform developed specifically for genome scaffolding with Omni-C data (Putnam *et al.* 2016). Dovetail Omni-C library sequences were aligned to our ONT-10× draft assembly using BWA (Li and Durbin 2009, 2010; Li 2013). The separations of Dovetail Omni-C read pairs mapped within draft scaffolds were analysed by HiRise to produce a likelihood model for genomic distance between read pairs, and the model was used to identify and break putative misjoins, to score prospective joins, and make joins above a threshold.

We screened our genome assembly for potential contamination with taxon-annotated GC-coverage plots using BlobTools (v.2.0; Laetsch and Blaxter 2017). To prepare data for BlobTools, we mapped both ONT and 10× raw reads against the final genome assembly using minimap2 (Li 2018) with the *-ax map-ont* and the *-ax sr* options, respectively. The resulting bam files were then sorted and merged with Samtools *sort* and *merge* commands (Li *et al.* 2009). A reference database for taxonomic assignment of scaffolds was created with MegaBLAST (Zhang *et al.* 2000) using the following parameters: -task megablast and -e-value 1e-25. We used the BlobTools module *map2cov* to calculate coverage and generated a database with the BlobTools command *create*. BlobTools results were visualized and plotted with the BlobTools command *view*. For each of the intermediate and the final assemblies, we produced genome contiguity and summary statistics using Assembly_Stats (v.0.14; Trizna 2020) and ran Benchmarking Universal Single Copy Orthologs (BUSCO v.3.0.2; Simão *et al.* 2015; Waterhouse *et al.* 2018; Manni *et al.* 2021) to assess the completeness of the slender anole genome final assembly. We scanned all the sequences for a vertebrate-specific database of 5,310 conserved genes (tetrapoda_odb10). We also estimated assembly completeness and consensus quality value (QV) by counting *k*-mers in short insert, 10× Illumina data using meryl (v1.3) with a *k*-value of 21 and inputting the meryl database, along with the final version of the assembly, to Merqury (v1.3; Rhie *et al.* 2020). Finally, we performed a conserved synteny analysis with the D-GENIES web-based software (Cabanettes and Klopp 2018), using minimap2 for alignment. We visualized synteny between our slender anole genome and the previously assembled slender anole genome (Tollis *et al.* 2018), as well as the green (Alföldi *et al.* 2011) and brown (Geneva *et al.* 2022) anole genomes. The final assembly statistics for our slender anole genome are presented in Table 1. All analyses were run using the Smithsonian Institution High Performance Computing Cluster.

**Table 1.** Descriptive statistics for the genome assemblies of the green anole (*A. carolinensis*; Alfoldi *et al.* 2011), the brown anole (*A. sagrei*; Geneva *et al.* 2022), the previous assembly of the slender anole (*A. apletophallus* Tollis *et al.* 2018), and the current assembly of the slender anole (this study).

	*A. carolinensis*		*A. sagrei*		*A. apletophallus*		*A. apletophallus*	
	Alfoldi *et al.* (2011)		Geneva *et al.* (2022)		Tollis *et al.* (2018)		Current study	
	Unplaced contigs	Scaffold	Unplaced contigs	Scaffold	Unplaced contigs	Scaffold	Unplaced contigs	Scaffold
L50 (number)	6,216	4	2,627	3	206,073	53,667	1,063	6
N50 (bp)	79,867	150,641,573	208,531	253,587,442	2,534	9,520	634,366	154,613,287
Longest (bp)	582,047	263,920,458	1,752,901	355,360,412	110,998	217,008	4,512,383	217,456,779
Median (bp)	21,470	10,682	8,573	1,348	—	—	17,299	9,783
Scaffold_count	41,987	6,457	32,431	3,738	—	—	17,399	9,445
Results	13 scaffolds > 0.25 Mb		14 scaffolds > 20 Mb		103 scaffolds > 100 kb		23 scaffolds > 4 Mb	
Total size (Gb)	1.89		1.93		2.18		2.4	

### Genome annotation

We annotated our slender anole genome using the Dovetail Genomics annotation pipeline from Jarvis *et al.* (2017). Briefly, this pipeline performs 6 steps. First, we applied repeat preparation and masking, which constructs a species-specific repeat model based on our genome assembly. Repeat families were identified de novo and classified using the software package RepeatModeler (version 2.0.3). RepeatModeler depends on the programs RECON (version 1.08) and RepeatScout (version 1.0.6) for the de novo identification of repeats within the genome. The custom repeat library obtained from RepeatModeler was used to discover, identify, and mask the repeats in the assembly file using RepeatMasker (Version 4.1.0). Second, we performed Model Preparation, which develops a species-specific hidden Markov model (HMM) that describes how genes are encoded. Here, we used coding sequences from *A. carolinensis*, *Lacerta agilis*, and *Zootoca vivipara* to train the initial ab initio model for the slender anole using the AUGUSTUS software (version 2.5.5). Six rounds of prediction optimization were done with AUGUSTUS. The same coding sequences were also used to train a separate ab initio model for the slender anole using SNAP (version 2006-07-28). Third, we used evidence preparation to generate gene structure based on transcriptome data (PRJNA961208), which we obtained from the liver, muscle, and brain tissue used in another study (Adam A. Rosso AA, Logan ML, Casement B, Chung AK, Curlis JD, Folfas E, Gallegos MA, Neel LK, Nicholson DJ, Williams CE, McMillan WO, Cox CL, personal comunication). RNA-seq reads were mapped onto the genome using the STAR aligner software (version 2.7; Dobin *et al.* 2013) and intron hints generated with the bam2hints tools within the AUGUSTUS software. MAKER, SNAP, and AUGUSTUS (with intron–exon boundary hints provided from RNA-seq) were then used to predict genes in the repeat-masked reference genome. To help guide the prediction process, Swiss-Prot peptide sequences from the UniProt database were downloaded and used in conjunction with the protein sequences from *A. carolinensis*, *L. agilis*, and *Z. vivipara* to generate peptide evidence in the MAKER pipeline. Only genes that were predicted by both SNAP and AUGUSTUS were retained in the final gene sets. To assess the quality of gene prediction, Annotation Edit Distance (AED) scores were generated for each of the predicted genes as part of the MAKER pipeline. Fourth, we curated the genome manually, during which we verified and corrected a preselected list of genes. We preselected genes that are biologically relevant for our focal species and our current research, such as arginyl–tRNA synthetase (RARS), heat shock protein family (HSP40), heat shock protein family A (HSP70), and the heat shock protein 90 (HSP90). Lastly, genes were further characterized for their putative function by performing a BLAST search of the peptide sequences against the UniProt database. tRNA were predicted using the software tRNAscan-SE (version 2.05).

### Repetitive element content and evolution

We estimated the repeat element (RE) composition and repetitive landscape of the slender anole genome using the RepeatModeler (v.2.03; Flynn *et al.* 2020) and RepeatMasker (v.4.1.2; Nishimura 2004) pipelines. We first constructed a reference repeat database for the slender anole by combining a de novo repeat library obtained from RepeatModeler and the *Anolis* repeat library from Repbase (release 20220927; Bao *et al.* 2015). We then annotated the repeats in our final assembly using RepeatMasker (v4.1.2). To estimate evolutionary divergence within repeat families, we generated a specific repeat family alignment and estimated the average Kimura-2-parameter divergence from consensus within each family while correcting for high mutation rates at CpG sites using the perl tool (calcDivergenceFromAlign.pl) from the RepeatMasker package. We compared the slender anole’s RE composition and divergence profile to the green anole (AnoCar2.0; Alföldi *et al.* 2011) and brown anole (AnoSag2.1; Geneva *et al.* 2022) assemblies using a parallel analysis with RepeatModeler and RepeatMasker.

## Results and discussion

### Genome assembly and comparison with other anoles

We assembled and annotated a highly contiguous genome for the slender anole through multiple rounds of improvement based on Oxford Nanopore (ONT), 10× Linked-Read technologies (Illumina), and Hi-C data (Dovetail). Based solely on the 10× Illumina data and GenomeScope results, the slender anole genome size was estimated to be 1.7 Gb (smaller than our final genome assembly) with approximately 71.4% unique content and a heterozygosity level of 1.35% (Supplementary Fig. 1). Although our initial hybrid assembly using Illumina 10× and ONT was fragmented, it produced a 2.4 Gb genome with a contig N50 of 428 kb, and the longest read was 4.5 Mb (see https://github.com/renatapirani/Genome-Anolis-apletophallus). The hybrid assembly also resulted in a total of ∼13.5 million ONT reads, constituting ∼73 Gb of sequence data, with an average read length of 5,458 kb (Supplementary Table 1). 10× read technologies generated close to ∼720 million paired-end reads, which produced ∼115 Gb of sequence data with an average cleaned read length of 148.5 bp. The third approach, Hi-C technologies, produced ∼144 million reads and ∼60.8 Gb of data, with an average read length of 300 bp (Supplementary Fig. 2). These data represent an approximate genome coverage of 30×, 44×, and 19×, respectively, based on our final genome assembly. The sequence data are summarized in detail in Supplementary Table 1. We used Hi-C data for genome scaffolding to enhance our initial draft assembly with a mapping rate of 99%. The scaffolding performed in HiRise using Hi-C proximity ligation libraries resulted in an improved assembly with the highest estimated gene representation. A comparison of the genome assembly obtained here with the previously assembled slender anole genome (Tollis *et al.* 2018) indicated that our genome had higher sequencing coverage, lower heterozygosity, and much higher contiguity and completeness (scaffold N50 of 154.6 kb in our final genome compared to 9.52 kb obtained; Tollis *et al.* 2018; Table 1).

Our assessment of contamination in BlobTools indicated that 72.4% (7,000 scaffolds, ∼2.4 Gb) of the scaffolds were classified as Eucaryota, whereas only 0.02% of the scaffolds mapped to bacteria (2 scaffolds). The remaining scaffolds (which represented a small total portion of the genome) had no blast hits (27.6%, 2,500 scaffolds, 18 Mb; Supplementary Fig. 3). At the genus level, 52.5% of reads mapped to anoles and 18.4% to other Chordata. The 2 microbial taxa present in the assembly were identified to genera *Bacillus* (0.01%) and *Ruegeria* (0.01%). *Bacillus* is ubiquitous in nature, and some can be pathogens in vertebrates. *Ruegeria* is aerobic and can be found on rhizosphere soil, so it is likely that this taxon was a contaminant present on the substrate on which our sample was collected.

**Fig. 2.**
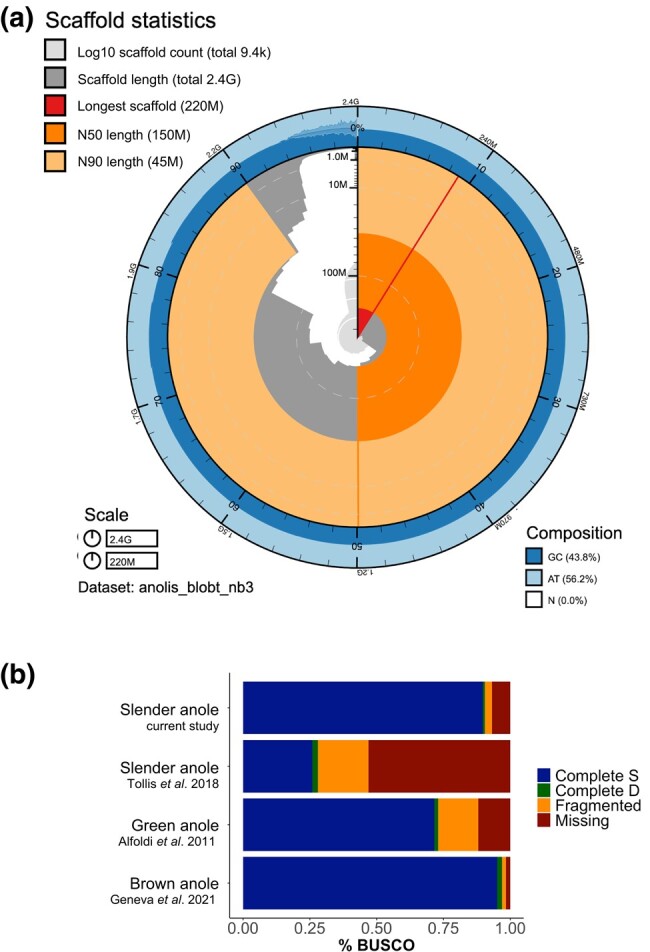
Genome assembly statistics for the slender anole and BUSCO completeness comparisons between anole genomes. a) Snail plot (produced using BlobTools v2.0) showing genome assembly summary statistics for our slender anole genome, including the scaffold N50 (dark orange), N90 (light orange), and base composition (percentage GC in dark blue, AT in light blue, and N in white). b) BUSCO results for the brown anole, green anole, and both the previous and current slender anole genome assemblies, including the portions of each genome that are composed of sequences that are complete and single copy (S), complete and duplicated (D), fragmented (F), or missing (M). These results are broken down in more detail in Supplementary Table 2.

Our final genome assembly for the slender anole was ∼2.4 Gb in size with a GC content of 43.8% (Fig. 2a). The slender anole genome is thus substantially larger than both the green anole (1.89 Gb; Alföldi *et al.* 2011) and brown anole (1.93 Gb; Geneva *et al.* 2022) genomes (Table 1). BUSCO (Manni *et al.* 2021) analysis using the Tetrapoda gene set recovered 90.5% of expected complete orthologs within our assembly, while in the previous slender anole genome, only 28% of the expected complete orthologs were recovered (Fig. 2b). Compared with the other high-quality (chromosome-level) anole genomes available on GenBank (*A. carolinensis* and *A. sagrei*), our assembly had the second highest scaffold N50, L50, and complete BUSCO scores (after the brown anole genome; Geneva *et al.* 2022), as well as the third fewest number of scaffolds (after the brown and green anole genomes; Fig. 2; Supplementary Table 2). Merqury estimated that assembly completeness was 92.5%, and the consensus QV score was 31 (>99.9% accuracy; Supplementary Fig. 4).

Dot plots comparing the slender, green, and brown anole assemblies show a high level of similarity between the 3 genomes (Supplementary Fig. 5). The first 10 scaffolds in the slender anole genome are likely the first 6 chromosomes present in the green anole genome (Alföldi *et al.* 2011; Eckalbar *et al.* 2013; Supplementary Fig. 5a). We observed a higher level of synteny between the slender and brown anoles, which we expected as both are part of the *Norops* species group (Supplementary Fig. 5b). Nevertheless, the macrochromosomes (5 largest) for the brown anole correspond to 10 chromosomes in the slender anole (Supplementary Fig. 5b). These results suggests that, despite generally high colinearity between the brown and slender anole genomes, there have been chromosome rearrangements that have taken place during the evolution of the *Norops* lineage.

### Genome annotation

Our annotation using the Dovetail pipeline identified a total of 46,763,836 bp coding regions and a total of 33,912 gene models. Protein-coding genes were 1,390 bp in length on average, with a total number of single-exon genes of 2,998. The number of gene models identified for the slender anole was higher than that of both the green anole (22,292) and brown anole (20,033). Dovetail functional annotation assigned putative functional predictions to 71% (24,182 genes) of the predicted protein-coding genes, leaving a total of 9,730 genes of unknown function.

### Repetitive element content and evolution

**Fig. 3.**
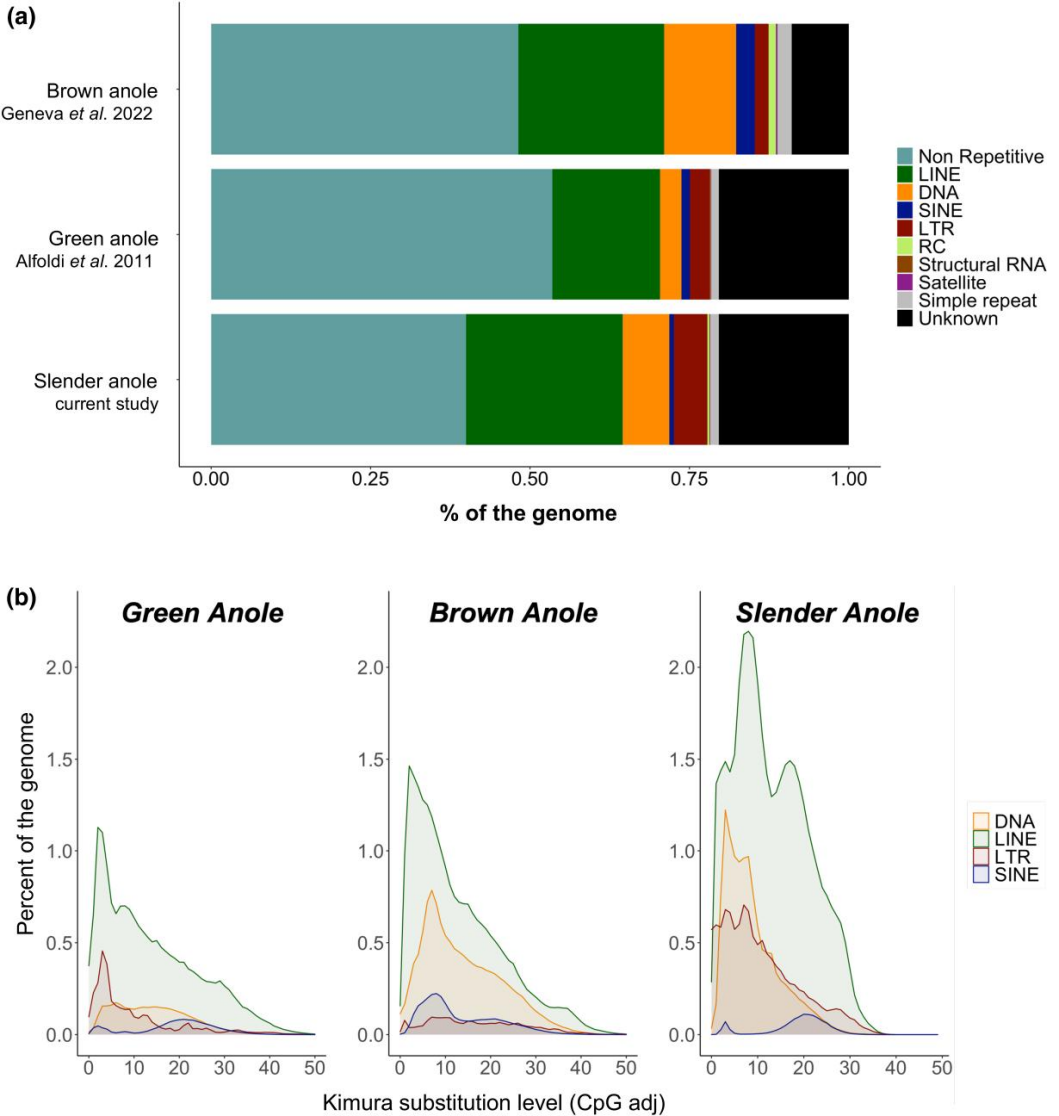
Proportional and landscape comparisons of RE content between 3 high-quality anole genomes. a) The proportion of REs of various categories (LINE, long interspersed elements; DNA, DNA transposons; SINE, short interspersed elements; LTR, long terminal repeat; RC, repetitive components, structural RNA, satellite, simple repeat, and unknown portions of the genome) across the 3 anole genomes. b) RE landscapes between the green, brown, and slender anole genomes according to their Kimura 2-parameter divergence from consensus.

We observed some clear differences in the composition of REs among anole species. In general, we estimated a total of 60% of the slender anole genome as REs, compared with 46.5% for the green anole and 51.8% for the brown anole (Fig. 3a). The slender anole genome had a greater proportion of long interspersed elements (LINEs; 24.5%) and long terminal repeat (LTR) retrotransposon elements (5.2%) compared with the green (16.8 and 3.3%, respectively) and brown anole (22.8 and 2.1%, respectively). In contrast, DNA transposon content was higher in the brown anole (DNA; 11.3%) compared with the slender and green anole (7.3 and 3.3%, respectively). A larger abundance of unknown REs were also recovered for the slender (20.3%) and green anole (20.3%), but a smaller fraction was detected in the brown anole (8.9%, Fig. 3a). Other RE classes and their proportions are included in Fig. 3a. There were also clear differences in RE content and evolution among the different anole lineages (Fig. 3b). Although a more accurate and complete RE annotation is required to make inferences about the evolution of REs in anoles, our current annotation and divergence analysis is useful to detect global patterns of RE activity between anole linages (Platt *et al.* 2016). The distributions of RE divergence in the slender anole reflect high divergence of all RE types with multiple peaks, which suggest evolution of REs has occurred both recently and more distantly in the past (multiple peaks between ∼2 and 20% divergence for all RE types; Fig. 3b). This contrasts with the pattern observed in the green and brown anoles, where only 1 peak was observed between ∼2 and ∼10% of divergence for all RE types, suggesting a more recent expansion of REs within those species (see also Feiner 2019). These differences between RE accumulation among anole species may be associated with differences in adaptation to alternative habitats (Kanamori *et al.* 2022) or demographic histories (Tollis *et al.* 2018).

### Conclusion

The genome of the slender anole described in this paper is one of the most complete and well-annotated genomes of any lizard species to date. This new genome provides a foundation for future studies on the genetic underpinnings of slender anole morphology, physiology, and behaviour and opens the door to develop this species as a model system for linking ecological-based selection to molecular evolution. The slender anole genome also increases the genetic resources available to study the evolution of mainland anoles and to improve our understanding of the adaptive radiation of this genus across the Neotropics.

## Supplementary Material

jkad248_Supplementary_Data

## Data Availability

The genome assembly and all the sequencing data have been deposited in the GenBank database under the accession number PRJNA906575. All supporting data and materials are available in the Smithsonian Tropical Research Institute repository (https://smithsonian.figshare.com/articles/dataset/Anolis_aplentophallus_genome_assembly_and_annotation/24352711). The codes for the analyses performed here can be found online at the GitHub repository (https://github.com/renatapirani/Genome-Anolis-apletophallus). Supplemental material available at G3 online.
