# Supplementary material for: A high-quality genome for the slender anole (Anolis apletophallus): an emerging model for field studies of tropical ecology and evolution
Source: G3 (Bethesda). 2023 Oct 24;14(1):jkad248. doi: 10.1093/g3journal/jkad248 (PMC10755174; doi:10.1093/g3journal/jkad248)
Supplement: jkad248_Supplementary_Data [file jkad248_supplementary_data.docx]

**A high-quality genome for the slender anole (*Anolis apletophallus*), an emerging model for field-studies of tropical ecology and evolution**

Renata M. Pirani, Carlos F. Arias, Kristin Charles, Albert K. Chung, John David Curlis, Daniel J. Nicholson, Marta Vargas, Christian L. Cox, W. Owen McMillan, Michael L. Logan

**Supplementary Material**

**Figure S1:** The histogram genome scope profile for the slender anole.

**Figure S2:** Density histogram plot from Dovetail Omni-C genome assembly.

**Figure S3:** Slender anole cumulative assembly span plot.

**Figure S4**: Merqury copy number spectrum plotted as stacked histogram.

**Figure S5:** D-GENIES dot plot visualization between anoles species.

**Table S1:** Summary table with the total number of reads and sequence coverage.

**Table S2:** BUSCO comparative results between different anole species.

## Figure S1: The Genomescope profile for the slender anole *Anolis apletophallus* before assembly. The kmer count distribution of the 10X Genomics linked‐reads (10X Genomics Chromium platform) was used to estimate the genome size.

**
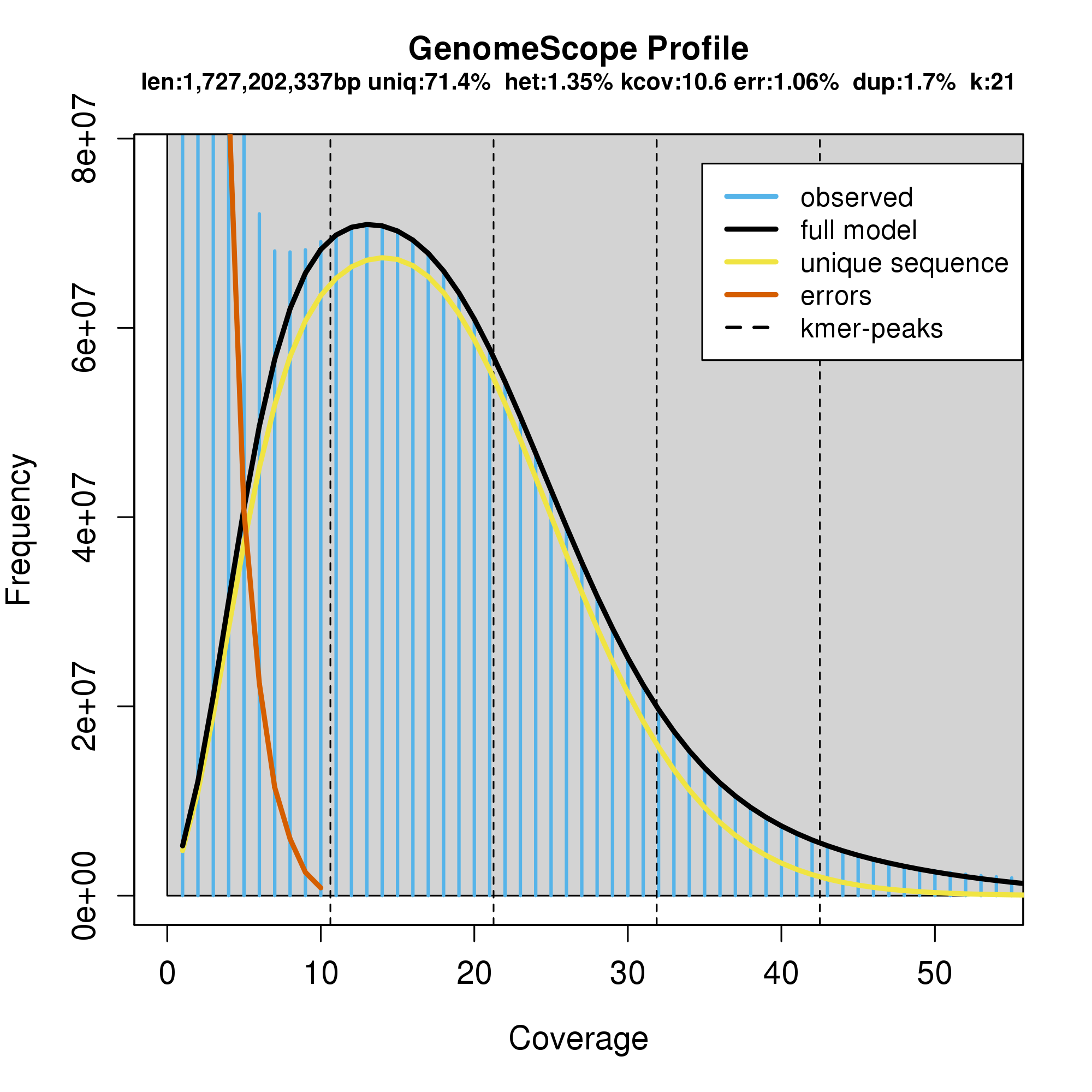
**

## Figure S2: Density histogram plot from Dovetail Omni-C genome assembly presenting the mate positions and read position from the slender anole genome.

##
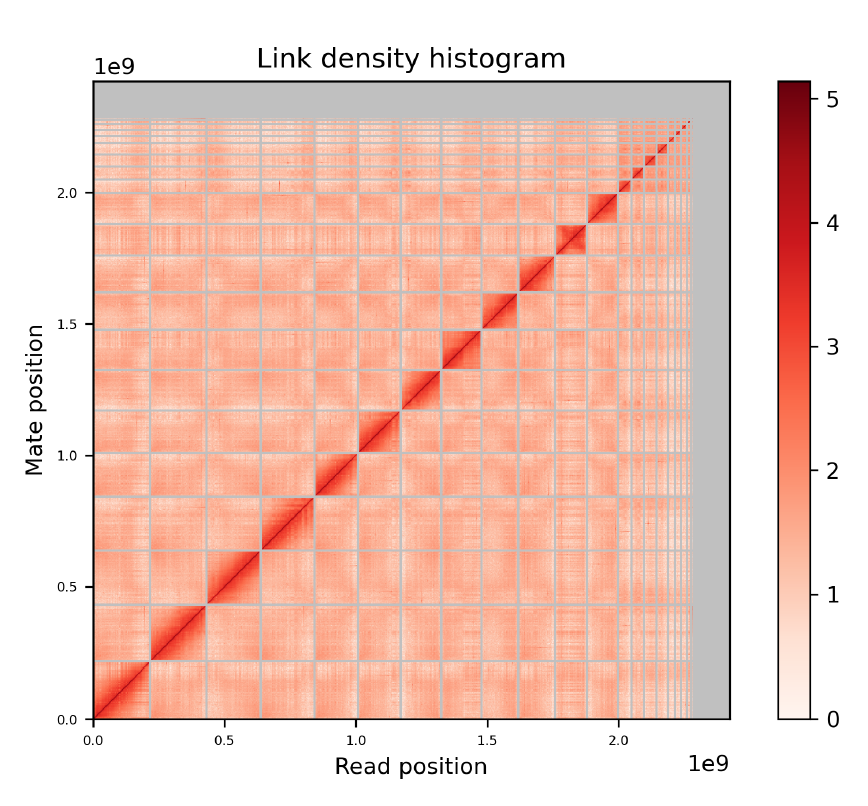


## Figure S3: Slender anole cumulative assembly span plot showing curves for subsets of scaffolds assigned to each phylum relative to the overall assembly.


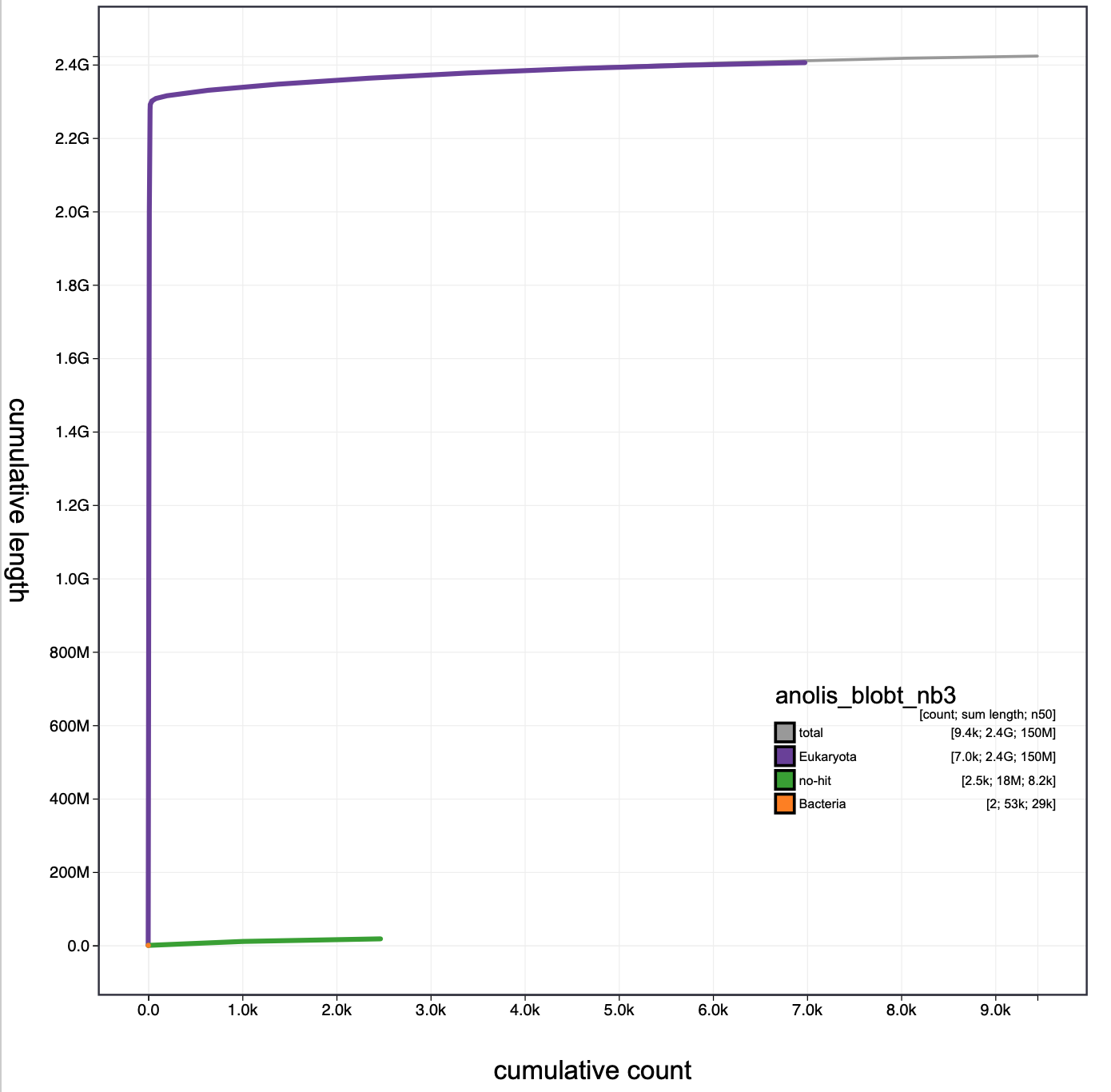


**Figure S4:** Merqury copy number spectrum plotted as stacked histogram colored by the k-mers numbers found on the slender anole genome assembly. The slender anole genome assembly presented an assembly completeness of 92.5% and the consensus QV score of 31 (>99.9% accuracy).


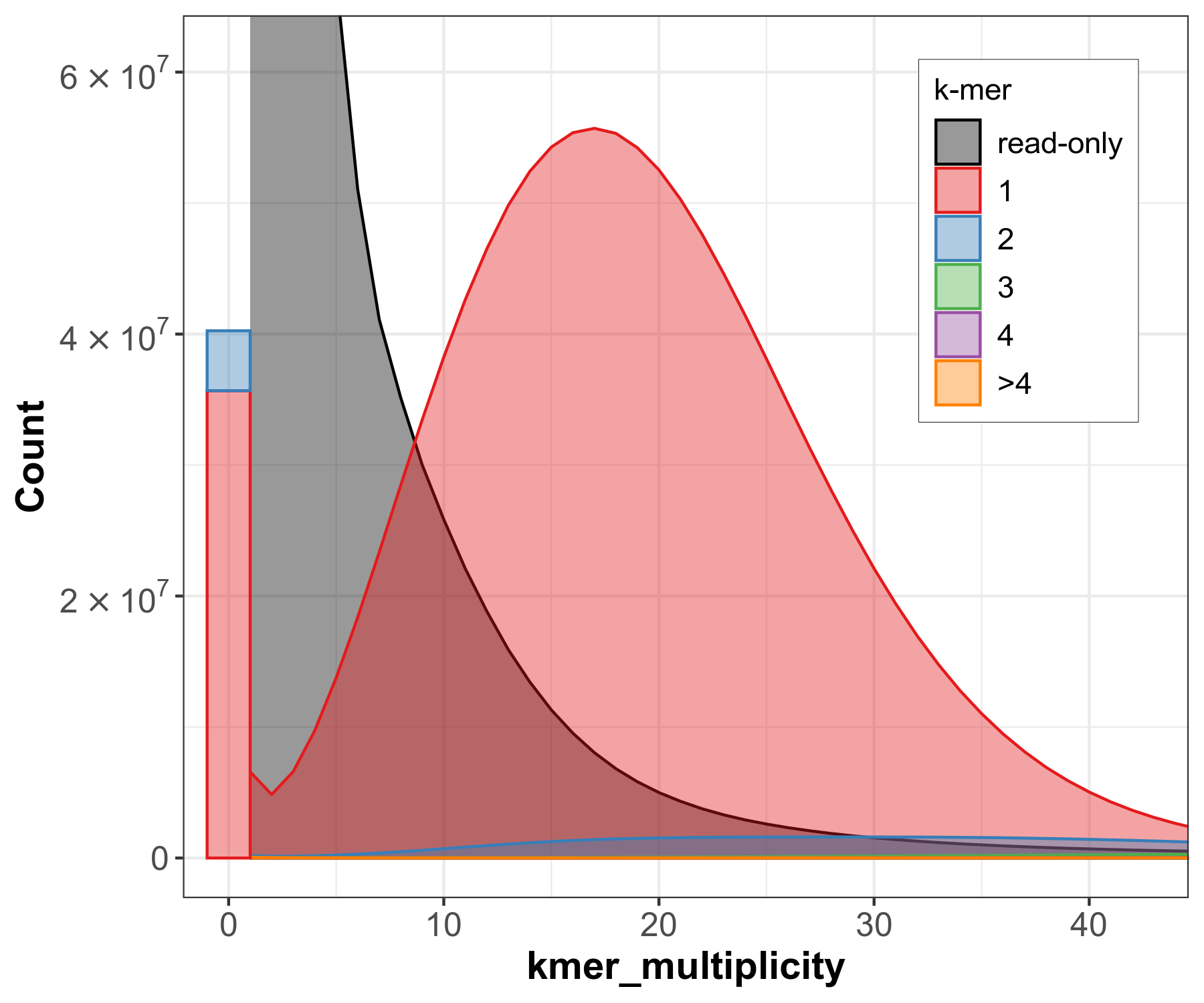


## Figure S5: D-GENIES dot plot visualization between anoles species. (A) green anole (top) and (B) brown anole (top) in the y-axis with the slender anole (right) presenting the growth of scaffold length ordered from the largest to smallest size on the x-axis for the slender anole. The plot (C) is the comparison between the green anole (top) and brown anole (right).

## Table S1: Summary table with the total number of reads and sequence coverage resulted from each technology used in this study. * It is the summary of read 1 = 360,062,312 and read 2 = 360,062,312 with base pairs of 53,090,862,465 and 52,626,048,857 respectively.

##

|  | **Oxford Nanopore (ONT)** | **10X Genomics** | **Dovetail Omni-C** |
| --- | --- | --- | --- |
| **Total number of reads** | 13,549,620 | 720,124,624* | 144,941,221 |
| **Average read length (pb)** | ~5,458 | 148.5 | 300 |
| **Gigabases of data (Gbp)** | 74 | 105.7 | 43.5 |
| **Sequence Coverage (pb)** | 58.9113913 | 46.3384541 | 18.7826087 |
| **Round Coverage** | ~30x | ~44x | ~19x |

## Table S2: BUSCO comparative results between different anole species. The table presents the assembled completeness comparison between green anole, brown anole, and both the previous and current slender anole genome assemblies.

|  | **Green anole** | **Brown anole** | **Slender anole** | **Slender anole** |
| --- | --- | --- | --- | --- |
|  | Alfoldi *et al*. 2011 | Geneva *et al*. 2021 | Tollis *et al*. 2018 | Current study |
| **Complete (C)** | 73% | 96.8% | 28% | 90.5% |
| **Complete and single-copy (S)** | 71.6% | 95.3% | 25.9% | 89.7% |
| **Complete and duplicated (D)** | 1.4% | 1.8% | 2.1% | 0.8% |
| **Fragmented (F)** | 15% | 1.5% | 19% | 2.7% |
| **Missing (M)** | 12% | 1.6% | 53% | 6.8% |
